# Comparison of bronchial brushing and sputum in detection of pediatric pulmonary tuberculosis

**DOI:** 10.1186/s13052-016-0221-3

**Published:** 2016-01-27

**Authors:** Qiao-pei Chen, Shi-feng Ren, Xin-feng Wang, Mao-shui Wang

**Affiliations:** Department of Clinical Laboratory, Guangxi Maternal and Child Health Hospital, Nanning, Guangxi PR China; Department of Lab Medicine, Xingming Hospital, Lanling, Shandong PR China; Department of Lab Medicine, Shandong Provincial Chest Hospital, 46# Lishan Road, Jinan, 250013 Shandong PR China

**Keywords:** Bronchial brushing, Sputum, Pediatric pulmonary tuberculosis, Diagnosis

## Abstract

The retrospective study aimed to evaluate the diagnostic value of bronchial brushing and sputum using acid fast bacilli smear, mycobacterial culture and real-time PCR in detection of pediatric pulmonary tuberculosis, sensitivity and specificity of bronchial brushing and sputum examined by the three methods were calculated and compared to each other. Data showed there were no significant difference in sensitivity between bronchial brushing and matched sputum using each method. But the specificity of real-time PCR on bronchial brushing was lower than on sputum. Compared with bronchial brushing, sputum was better specimen in detection of pediatric pulmonary tuberculosis.

## Dear editor,

Pediatric tuberculosis (TB) is often not considered a priority by national TB control programs, because the paucibacillary nature makes limited contribution to TB transmission. WHO estimated that in 2012 there were 530 000 TB cases among children and 74 000 deaths among HIV-negative children [1]. Diagnosing pediatric TB is challenging, because of non-specific symptoms, difficulties in obtaining samples for microbiological examination and paucibacillary nature of their disease. Bronchial brushing is commonly used in human medicine as a diagnostic tool to sample neoplastic lesions or for microbiological analysis [2]. In the retrospective study, the role of bronchial brushing for diagnosing pediatric pulmonary TB was evaluated while compared with of sputum.

## Materials and methods

Between Jul, 2007 and Apr, 2015, bronchial brushing via the flexible fiberoptic bronchoscope (Erbokryo CA, Erbe, Germany) and matched sputum were examined by acid fast bacilli (AFB) smear (Auramine O stain), mycobacterial culture (Lowenstein Jensen medium) and real-time PCR (Polymerase chain reaction) (TB RT-PCR kit, DAAN, China) in 59 pediatric patients (<15 years old). 8 patients were excluded for uncertain diseases. The reaming 51 patients (12.3 ± 1.4 years old, 26 male) with determined diseases were enrolled, and then were divided into pulmonary TB group (27 cases, 12.7 ± 1.3 years old, 12 male) and control group (24 cases, 11.8 ± 1.4 years old, 14 male). Pediatric pulmonary TB patients were diagnosed based on TB contact, clinical symptoms (fever, anorexia, weight loss, cough and a response to anti-tuberculosis therapy), chest X-ray examination, tuberculin skin test and routine TB assays. Patients were classified as control subjects when an alternative diagnosis was established. Differences in sensitivity and specificity between specimens were estimated by the McNemar’s test, with *P* < 0.05 considered significant. All calculations were estimated at a 95 % confidence interval (95 % CI). The protocol was approved by the Ethical Committee of the institute. Written informed consent was waived because of the retrospective nature.

## Findings

As shown in Table 1, the specificity (100 %, 95 % CI: 86.2–100 %) of AFB smear, mycobacterial culture and combination of the three tests on bronchial brushing was equal (all *P* > 0.05) with on sputum (100 %, 95 % CI: 86.2–100 %), but the specificity of real-time PCR (91.7 %, 95 % CI: 74.2–97.7 %) on bronchial brushing was lower than on sputum (100 %, 95 % CI: 86.2–100 %) (*P* < 0.01). For detection of pediatric pulmonary TB, sensitivities of AFB smear, mycobacterial culture, real-time PCR, or combination of the three tests on both specimens were, (1) bronchial brushing, 29.6 % (95 % CI: 15.9–48.5 %), 37.0 % (95 % CI: 21.5–55.8 %), 33.3 % (95 % CI: 18.6–52.2 %) and 48.2 % (95 % CI: 30.7–66.0 %), respectively; (2)sputum, 33.3 % (95 % CI: 18.6–52.2 %), 44.4 % (95 % CI: 27.6–62.7 %), 33.3 % (95 % CI: 18.6–52.2 %) and 55.6 % (95 % CI: 37.3–72.4 %), respectively. McNemar’s tests showed there were no significant difference in sensitivity between bronchial brushing and matched sputum using each method (all *P* > 0.05).

**Table 1:** Performance of the both specimens using TB assays in detection of pediatric pulmonary TB

	Sensitivity (95 % CI)		Specificity (95 % CI)	
	Bronchial brushing	Sputum	Bronchial brushing	Sputum
AFB smear	29.6 % (15.9–48.5 %)	33.3 % (18.6–52.2 %)	100 % (86.2–100 %)	100 % (86.2–100 %)
Mycobacterial cutlrue	37.0 % (21.5–55.8 %)	44.4 % (27.6–62.7 %)	100 % (86.2–100 %)	100 % (86.2–100 %)
Real–time PCR	33.3 % (18.6–52.2 %)	33.3 % (18.6–52.2 %)	91.7 % (74.2–97.7 %)	100 % (86.2–100 %)
Combination	48.2 % (30.7–66.0 %)	55.6 % (37.3–72.4 %)	100 % (86.2–100 %)	100 % (86.2–100 %)

*CI* confidence interval, *AFB* acid-fast bacilli, *PCR* polymerase chain reaction

## Discussion

Pediatric TB is difficult to diagnose, and efforts are made to improve its diagnostic accuracy, including trying different specimens, such as bronchoalveolar lavage fluid (BALF), induced sputum, nasopharynx swab [3]. Unfortunately, routine technologies performed on them cannot detect pediatric TB accurately [4]. Currently, few studies have been performed aiming to evaluate the diagnostic role of bronchial brushing in detection of pulmonary TB. Mehta J et al found that smears of brush/wash specimens from fiberoptic bronchoscopy were of comparable sensitivity to those of sputum [5]. In our study, we showed the similar results. Meanwhile, mycobacterial culture and real-time PCR performed on bronchial brushing or sputum also have same sensitivities in detection of pediatric TB. We also compared BALF and sputum in detection of pediatric pulmonary TB (data not published). The three assays were all negative in BALF and sputum among Non-pulmonary TB patients (three cases); among the six pulmonary TB patients, three were BALF culture positive, one was real-time PCR positive in BALF, no one was positive in sputum using these assays. The results showed that the BALF tended to be better than sputum in detection of pediatric pulmonary TB in the help of the three assays. Since few cases (only three) were examined parallel in BALF and bronchial brushing using the three assays, further comparison of BALF, sputum and bronchial brushing weren’t conducted. The limitations of the study included: (1) retrospective nature; (2) It was difficult to assess the utility of induced sputum and BALF, as they were performed in few children; (3) because of younger children usually have difficulty in producing a sputum, the enrolled subjects all aged >10 years old.

In conclusion, compared with bronchial brushing, sputum was better specimen in detection of pediatric pulmonary TB when using AFB smear, mycobacterial culture and real-time PCR.

## Abbreviations

BALF: bronchoalveolar lavage fluid
PCR: polymerase chain reaction
TB: tuberculosis
95 % CI: 95 % confidence interval
